# Underweight and overweight/obesity among adults in Afghanistan: prevalence and correlates from a national survey in 2018

**DOI:** 10.1186/s41043-021-00251-0

**Published:** 2021-06-06

**Authors:** Supa Pengpid, Karl Peltzer

**Affiliations:** 1 ASEAN Institute for Health Development, Mahidol University, Salaya, Phutthamonthon, Nakhon Pathom, Thailand; 2 Department of Research Administration and Development, University of Limpopo, Turfloop, Mankweng, South Africa; 3 Department of Psychology, University of the Free State, Bloemfontein, South Africa; 4 Department of Psychology, College of Medical and Health Science, Asia University, Taichung, Taiwan

**Keywords:** Body weight, Health behaviour, Health status, Adulthood

## Abstract

**Background:**

The study aimed to estimate the prevalence and correlates of underweight and overweight/obesity among adults in Afghanistan.

**Methods:**

National cross-sectional survey data of 3779 persons aged 18–69 years were analysed. Multinomial logistic regression was used to estimate factors associated with underweight and overweight/obesity relative to normal weight.

**Results:**

In all, 7.8% of the study sample was underweight (BMI < 18.5 kg/m^2^), 49.5% had normal weight (BMI 18.5–24.9 kg/m^2^), 25.5% overweight (25.0–29.9 kg/m^2^), and 17.2% obesity. In adjusted multinomial logistic regression, factors negatively associated with underweight were male sex (adjusted relative risk ratio (ARRR): 0.30, 95% confidence interval (CI): 0.15–0.58) and hypertension (ARRR: 0.51, 95% CI: 0.27–0.95) and factors positively associated with underweight were sedentary behaviour (ARRR: 1.85, 95% CI: 1.11–3.10) and current tobacco use (ARRR: 2.58, 95% CI: 1.08–6.16). Factors positively associated with overweight/obesity were aged 30–44 years (ARRR: 2.00, CI: 1.51–2.66) and aged 45–69 years (ARRR: 1.58, CI: 1.09–2.31) (compared to 18–29 years) (ARRR: 1.28, CI: 1.14–2.18), hypertension (ARRR: 2.74, CI: 1.89–3.96), and type 2 diabetes (ARRR: 1.82, CI: 1.13–2.94), and high physical activity (ARRR: 0.70, CI: 0.50–0.98) was negatively associated with overweight/obesity.

**Conclusion:**

Almost one in ten adult respondents were underweight and more than two in five were overweight/obese, confirming a dual burden of malnutrition in Afghanistan.

## Introduction

Worldwide, among adults, the prevalence of undernutrition (18.5 < kg/m^2^) was 8.8% among men and 9.7% among women, and the prevalence of obesity (BMI ≥ 30 kg/m^2^) was 10.8% among men and 14.9% among women [1]. In the Eastern Mediterranean region, among adults, a high average prevalence of overweight/obesity (51%) [2], ranging from 25 to 81.9% has been reported [3]. In Iran, the prevalence of underweight was 5.9% and 49.9% had overweight/obesity (15–65 years, in 2004–2005) [4]; in Iraq (≥ 15 years, in 2015), underweight was 3.6% and overweight/obesity 65.7% [5]; in Jordan, overweight or obesity (BMI ≥ 25 kg/m^2^) was 77.2% among men and 74.5% among women (≥ 18 years; 2017) [6]; in Morocco, underweight was 5.6% and overweight/obesity 56.1% (≥ 18 years; 2017) [7]; and in Palestine (18–64 years, 1999–2000), underweight was 1.5% and overweight/obesity 62.4% [8].

To our knowledge, there are no national adult body weight status data for Afghanistan. Afghanistan is a low-income country, its living standards are among the lowest in the world, it has a population of 36.6 million, life expectancy at birth was 52.8 years, and the adult literacy rate was 43% (55.5% among men and 29.8% among women) [9].

In local community surveys in urban centres in Afghanistan, the following proportions of body weight status have previously been shown: in Kabul City (≥ 40 years, in 2011–2012), underweight 1.1% and overweight/obesity 69.3% [10]; in Kabul (25–70 years, in 2015), 57.6% overweight/obesity [11]; in Jalalabad (25–65 years, in 2013) underweight 6.1% [12] and overweight/obesity 57.4% [13]; and in Kabul, Balkh, Hirat, Nangarhar, and Kandahar (25–70 years, during 2013–2015), overweight/obesity was 52.7% [14]. In a national study among women 15–49 years in Afghanistan, the prevalence of underweight was 8.6% [15].

Both undernutrition and overnutrition in adulthood have been linked to various negative health effects, including morbidity and mortality [16, 17]. As reviewed [18], sociodemographic factors associated with adult underweight may include female sex, younger and older age, lower socioeconomic status, and residing in rural areas. Health variables associated with adult underweight may include poor diets, smoking, and not having chronic conditions. As reviewed [18], sociodemographic factors associated with overweight/obesity include female sex, increasing age, higher socioeconomic status, and urban residence, and health variables associated with overweight/obesity may include poor diet, physical inactivity, not smoking, diabetes, and hypertension. Afghanistan has a high prevalence of undernutrition in children (19.1% in 2018) under the age of five [9, 15], which may affect adult weight status. The study aimed to estimate for the first time the national prevalence and correlates of underweight and overweight/among adults in Afghanistan.

## Methods

This is a secondary analysis conducted using nationally representative population-based and cross-sectional data from the “2018 Afghanistan STEPS survey” [19]. The 2018 Afghanistan STEPS survey data and more detailed sampling methods can be accessed [20]. Briefly, a multistage cluster sampling was used to generate a nationally representative sample of adults aged 18–69 years. Stage 1 or primary sampling units were 55 districts, followed by villages or blocks (secondary sampling units) and households (tertiary sampling units). One person from each household was randomly selected [20]. In total, 3956 persons aged 18–69 years were potentially eligible in this study. However, 177 women were excluded as they had indicated to be pregnant during the study so that 3779 participants were included in the final data analysis. The study instrument was translated to Pashto and Dari and piloted [20]. The study was approved by the Ministry of Public Health Ethics Board, and written informed consent was obtained from participants [20].

### Measures

Anthropometric measurements were taken by trained healthcare staff in a safe and secure area; height and weight were measured using a portable electronic weighing scale and measuring inflexible bars [20]. “Height in centimetres is measured at the exact point to the nearest mm. Participants were weighed to the nearest 0.01 kg, in their light clothes, on a load-cell-operated digital scale. The scale used during the survey were first calibrated to a standard weight and checked daily.” [20]. Body mass index (BMI) was classified as “< 18.5 kg/m^2^ underweight, 18.5–24.4 kg/m^2^ normal weight, 25–29.9 kg/m^2^ overweight, and ≥ 30 kg/m^2^ obesity” [21].

*Hypertension or raised blood pressure* (BP) was defined as “systolic BP ≥ 140 mm Hg and/or diastolic BP ≥ 90 mmHg or where the participant is currently on antihypertensive medication.” [22]. BP was measured with a calibrated sphygmomanometer after participants had been seated at least for 15 min, and three minutes in between readings. Of the three BP measurements, the last two readings were averaged [20].

*Diabetes* was defined as “fasting plasma glucose levels ≥ 7.0 mmol/L (126 mg/dl); or using insulin or oral hypoglycaemic drugs; or having a history of diagnosis of diabetes” [23].

*Health risk behaviour* variables comprised alcohol use, tobacco use status, sedentary behaviour (≥ 8 h/day [24]), and “low, moderate or high physical activity based on the Global Physical Activity Questionnaire” [25].

*Dietary behaviour* included “daily fruit and vegetable consumption measured from the total number of servings of fruit and vegetables eaten per day in a typical week.” Meals outside the home were assessed with the question, “On average, how many meals per week do you eat that were not prepared at home? By meal, I mean breakfast, lunch and dinner?” (Number) [20]. Adding salt to meals was measured with the item “How often do you add salt or a salty sauce such as soya sauce to your food right before you eat it or as you are eating it?” (Response options ranged from 1 = always to 5 = never). Eating processed food was assessed with the item “How often do you eat processed food high in salt? By processed food high in salt, I mean foods that have been altered from their natural state, such as packaged salty snacks, canned salty food including pickles and preserves, salty food prepared at a fast food restaurant, cheese, bacon and processed meat.” (Response options ranged from 1 = always to 5 = never). “What type of oil or fat is most often used for meal preparation in your household?” Responses were grouped into mainly butter or ghee (59.5%) (or lard or suet 0.3%, margarine 0.3%, none in particular 1.7% or none 0.0%) used = 0 and vegetable oil (38.2%) = 1 [20].

*Sociodemographic information* included age, sex, highest educational level, number of adult household members, and residence status [20]. Household crowding has been found to have an inverse relationship with socioeconomic status [26].

### Data analysis

All statistical procedures were adjusted for complex sample design and conducted with “STATA software version 13.0 (Stata Corporation, College Station, TX, USA)”. The data were weighted “to make the sample representative of the target population in Afghanistan (by sex and by age group: 18–29, 30–44, 45 and over).” [20]. Chi-square test calculated differences in proportions. Multivariable multinomial logistic regression was used to estimate predictors of underweight and overweight/obesity (with normal body weight forming the reference category). Covariates were included based on a previous review of literature [18]. No multi-collinearity was detected. Missing data were excluded from the analysis. *P* < 0.05 was considered significant.

## Results

### Sample and body mass index information

The sample consisted 3779 individuals aged 18–69 years (median age: 35 years, 24–48 interquartile range), and 55.9% were male. Two in five participants (41.8%) were living with six or more adult household members, 59.0% had no formal education, and 42.4% lived in rural areas. In all, 7.8% of the study sample was underweight (BMI < 18.5 kg/m^2^), 49.5% had normal weight (BMI 18.5–24.9 kg/m^2^), 25.5% overweight (25.0–29.9 kg/m^2^), and 17.2% obesity. Further sample details are shown in Table 1.

**Table 1:** Sample and nutritional status among adults in Afghanistan, 2018

Variable (#missing values)	Sample	Normal weight	Underweight	Overweight	Obesity	***p*** value
	N (%)	N (%)	N (%)	N (%)	N (%)	
**All**	3779	1774 (49.5)	264 (7.8)	1071 (25.5)	636 (17.2)	
Age in years (#30)						
18-29	1382 (44.1)	776 (58.8)	131 (8.9)	307 (21.0)	144 (11.3)	< 0.001
30-44	1124 (32.2)	460 (42.7)	69 (7.4)	352 (27.1)	239 (22.8)	
45-69	1243 (23.8)	525 (41.8)	62 (6.2)	402 (31.6)	252 (20.4)	
Sex (#4)						
Female	1753 (44.1)	723 (42.1)	156 (9.1)	465 (25.1)	389 (23.7)	< 0.001
Male	2022 (55.9)	1051 (55.3)	108 (6.7)	606 (25.9)	247 (12.1)	
Education (#3)						
None	2094 (59.0)	940 (46.9)	147 (6.4)	582 (26.8)	411 (20.0)	0.133
Primary or less	659 (16.4)	309 (53.0)	38 (8.5)	206 (23.1)	103 (15.4)	
Secondary or more	1023 (24.6)	525 (53.4)	79 (10.7)	283 (24.0)	122 (11.9)	
Adult household members (#3)						
1-3	1352 (23.9)	616 (50.5)	102 (7.2)	402 (28.7)	215 (13.7)	0.269
3-5	1229 (34.3)	606 (49.9)	90 (8.9)	347 (25.6)	178 (15.6)	
≥6	1195 (41.8)	552 (48.6)	72 (7.1)	322 (23.7)	243 (20.6)	
Residence (#1)						
Rural	1797 (42.4)	922 (51.9)	140 (8.3)	471 (25.1)	249 (14.7)	0.306
Urban	1981 (57.6)	852 (47.7)	124 (7.3)	600 (25.8)	387 (19.1)	
Fruit and vegetable consumption (#0)						
≤1 servings	2415 (59.0)	1164 (50.3)	182 (8.7)	650 (25.3)	401 (15.7)	0.394
2 servings	883 (29.6)	389 (47.9)	51 (6.8)	285 (27.8)	149 (17.5)	
≥3 servings	481 (11.4)	221 (49.4)	31 (5.5)	136 (20.9)	86 (24.2)	
For meal preparation (#6)						
Mainly butter or ghee	2048 (61.8)	980 (49.3)	142 (6.3)	546 (24.9)	365 (19.5)	0.022
Vegetable oil	1725 (38.2)	793 (49.9)	122 (10.1)	523 (26.3)	271 (13.7)	
Add salt before/when eating (#8)						
Sometimes or rarely or never	2697 (68.4)	1242 (48.1)	186 (8.6)	792 (26.5)	460 (16.8)	0.226
Always or often	1074 (31.6)	530 (52.5)	76 (5.8)	279 (23.5)	176 (18.2)	
Eats processed foods high in salt (#27)						
Sometimes or rarely or never	3077 (87.5)	1424 (49.5)	216 (7.8)	893 (25.6)	524 (17.0)	0.930
Always or often	675 (12.5)	338 (48.7)	47 (7.3)	173 (25.2)	107 (18.9)	
Meals outside home (#55)						
0	2477 (65.9)	1133 (47.0)	178 (8.3)	684 (25.7)	468 (19.0)	0.374
≥ 1	1247 (34.1)	610 (53.9)	79 (6.5)	376 (25.4)	376 (14.1)	
Physical activity (#35)						
Low	1384 (38.4)	591 (41.5)	94 (7.6)	377 (27.1)	303 (23.8)	< 0.001
Moderate	624 (18.3)	267 (50.9)	46 (7.3)	202 (24.4)	106 (17.4)	
High	1736 (43.3)	903 (56.0)	123 (8.1)	480 (24.6)	221 (11.4)	
Sedentary behaviour (#24)						
< 8 h/day	2128 (51.9)	1037 (54.5)	144 (6.6)	580 (23.9)	346 (15.1)	0.012
≥8 h/day	1627 (48.1)	725 (44.1)	120 (9.1)	486 (27.1)	286 (19.6)	
Current tobacco use (#3)						
No	2910 (72.0)	1348 (50.2)	207 (7.0)	825 (25.0)	502 (17.8)	0.557
Yes	866 (28.0)	426 (47.7)	57 (9.6)	246 (26.9)	134 (15.8)	
Ever alcohol use (#3)						
No	3732 (99.5)	1751 (49.4)	261 (7.8)	1059 (25.5)	630 (17.3)	0.835
Yes	44 (0.5)	23 (58.9)	3 (8.4)	12 (21.5)	6 (11.2)	
Hypertension (#40)						
No	2566 (69.3)	1376 (56.9)	218 (9.8)	667 (21.9)	304 (11.4)	< 0.001
Yes	1173 (30.7)	391 (32.6)	46 (3.1)	404 (33.8)	331 (30.4)	
Type 2 diabetes (#292)						
No	3083 (90.3)	1500 (52.2)	211 (7.7)	870 (25.1)	480 (14.9)	< 0.001
Yes	404 (9.7)	147 (27.3)	26 (4.8)	132 (31.4)	98 (36.6)	
Heart attack or stroke (#3)						
No	3488 (90.6)	1658 (49.6)	250 (7.9)	978 (25.5)	571 (17.1)	0.933
Yes	288 (9.4)	116 (48.8)	14 (6.5)	93 (25.9)	65 (18.7)	

### Multinomial logistic regression for underweight and overweight/obesity

In adjusted multinomial logistic regression, factors negatively associated with underweight were male sex (adjusted relative risk ratio (ARRR): 0.30, 95% confidence interval (CI): 0.15–0.58) and hypertension (ARRR: 0.51, 95% CI: 0.27–0.95), and factors positively associated with underweight were sedentary behaviour (ARRR: 1.85, 95% CI: 1.11–3.10) and current tobacco use (ARRR: 2.58, 95% CI: 1.08–6.16). Factors positively associated with overweight/obesity were aged 30–44 years (ARRR: 2.00, CI: 1.51–2.66) and aged 45–69 years (ARRR: 1.58, CI: 1.09–2.31) (compared to 18–29 years) (ARRR: 1.28, CI: 1.14–2.18), hypertension (ARRR: 2.74, CI: 1.89–3.96), and type 2 diabetes (ARRR: 1.82, CI: 1.13–2.94), and high physical activity (ARRR: 0.70, CI: 0.50–0.98) was negatively associated with overweight/obesity (see Table 2).

**Table 2:** Multivariable associations with underweight and overweight/obesity (with normal weight as reference category)

Variable	Underweight		Overweight/Obesity	
	Adjusted RRR (95% CI)	*p* value	Adjusted RRR (95% CI)	*p* value
Age in years				
18–29	1 (Reference)		1 (Reference)	
30–44	0.90 (0.55, 1.77)	0.972	2.00 (1.51, 2.66)	< 0.001
45–69	0.91 (0.45, 1.87)	0.805	1.58 (1.09, 2.31)	0.017
Sex				
Female	1 (Reference)		1 (Reference)	
Male	0.30 (0.15, 0.58)	< 0.001	0.74 (0.47, 1.17)	0.201
Education				
None	1 (Reference)		1 (Reference)	
Primary or less	2.01 (0.67, 6.02)	0.210	0.88 (0.58, 1.32)	0.528
Secondary or more	2.75 (0.98, 7.71)	0.054	1.06 (0.65, 1.74)	0.809
Adult household members				
1–3	1 (Reference)		1 (Reference)	
3–5	1.22 (0.66, 2.26)	0.533	0.82 (0.55, 1.20)	0.299
≥ 6	0.96 (0.52, 1.80)	0.908	0.93 (0.69, 1.31)	0.689
Residence				
Rural	1 (Reference)		1 (Reference)	
Urban	0.70 (0.42, 1.15)	0.156	1.05 (0.79, 1.40)	0.733
Fruit and vegetable consumption				
≤ 1 servings	1 (Reference)		1 (Reference)	
2 servings	0.85 (0.49, 1.46)	0.548	1.11 (0.69, 1.79)	0.670
≥ 3 servings	0.36 (0.09, 1.50)	0.159	1.26 (0.84, 1.90)	0.264
For meal preparation				
Mainly butter or ghee	1 (Reference)		1 (Reference)	
Vegetable oil	1.65 (0.82, 3.31)	0.157	0.99 (0.78, 1.27)	0.967
Add salt before/when eating				
Sometimes or rarely or never	1 (Reference)		1 (Reference)	
Always or often	0.48 (0.28, 1.03)	0.062	0.91 (0.64, 1.29)	0.590
Eats processed foods high in salt				
Sometimes or rarely or never	1 (Reference)		1 (Reference)	
Always or often	0.85 (0.44, 1.64)	0.620	1.36 (0.74, 2.50)	0.315
Meals outside home				
0	1 (Reference)		1 (Reference)	
≥ 1	0.73 (0.20, 2.64)	0.624	1.03 (0.57, 1.87)	0.923
Physical activity				
Low	1 (Reference)		1 (Reference)	
Moderate	0.72 (0.32, 1.64)	0.434	0.75 (0.51, 1.10)	0.143
High	0.77 (0.43, 1.38)	0.371	0.70 (0.50, 0.98)	0.037
Sedentary behaviour				
< 8 h/day	1 (Reference)		1 (Reference)	
≥ 8 h/day	1.85 (1.11, 3.10)	0.019	1.24 (0.95, 1.63)	0.112
Current tobacco use				
No	1 (Reference)		1 (Reference)	
Yes	2.58 (1.08, 6.16)	0.033	1.13 (0.83, 1.55)	0.439
Ever alcohol use				
No	1 (Reference)		1 (Reference)	
Yes	0.73 (0.08, 6.79)	0.783	0.41 (0.08, 2.08)	0.279
Hypertension				
No	1 (Reference)		1 (Reference)	
Yes	0.51 (0.27, 0.95)	0.034	2.74 (1.89, 3.96)	< 0.001
Type 2 diabetes				
No	1 (Reference)		1 (Reference)	
Yes	1.17 (0.53, 2.52)	0.704	1.82 (1.13, 2.94)	0.014

*RRR* relative risk ratio, *CI* confidence interval

## Discussion

In this national 2018 Afghanistan STEPS survey, the prevalence of underweight (7.8%) was higher than in Kabul City (1.1%, ≥ 40 years, 2011–2012) [10], in Jalalabad (6.1%, 25–65 years, in 2013) [12], in Iran (5.9%, 15–65 years, 2004–2005) [4], in Iraq (3.6%, ≥ 15 years, 2015) [5], in Morocco (5.6%, ≥ 18 years, 2017) [7], and Palestine (1.5%, 18–64 years, 1999–2000) [8], but similar to a national study among women in Afghanistan (8.6%, 15–49 years vs. 9.1%, 18–69 years, in this study) [15], and the global prevalence of underweight (8.8% among men and 9.7% among women) [1]. The found prevalence of overweight/obesity (42.7%, ≥ 25.0 kg/m^2^) in this study is lower than the prevalence rates found in urban centres in Afghanistan, e.g., in Kabul City (69.3%, ≥ 40 years, 2011–2012) [10], in Kabul (57.6%, 25–70 years, 2015) [11], in Jalalabad (57.4%, 25–65 years, 2013) [13], in Kabul, Balkh, Hirat, Nangarhar, and Kandahar (52.7%, 25–70 years, 2013–2015) [14], in Iran (59.3%, 2016) [27], in Iraq (65.7%, ≥ 15 years, in 2015) [5], in Morocco (56.1% ≥ 18 years, 2017) [7], Palestine (62.4%, 18–64 years, 1999–2000) [8], and in Jordan (> 75%, ≥ 18 years, 2017) [6].

Findings show the double burden of undernutrition (7.8%) and overnutrition (42.7%, ≥ 25 kg/m^2^) in the low-income country, Afghanistan. The co-existence of undernutrition (15.6%) and overnutrition (18.0%) has also been found in low-income countries in the Asia Pacific region [28]. The trend in the reduction of underweight and increase of overweight/obesity [1, 28] seems to have been confirmed in this study in Afghanistan. “Rapid dietary and lifestyle transition is the leading direction of dual burden with overnutrition increase and diet-related non-communicable diseases (NCDs)” [28, 29]. In addition, it is possible that the high prevalence of undernutrition in children under the age of five in Afghanistan [15] has led to increased overnutrition in adulthood [30]. Increased efforts on policy initiatives and lifestyle changes are needed in Afghanistan to combat the double malnutrition burden.

The prevalence of underweight was the highest among 18 to 29 year olds (8.9%) and among women (9.1%), which was also found in previous studies [31–33], and may be attributed to food insecurity, in particular among young women [15, 34]. Akseer et al. [15] showed that younger adolescent mothers (< 20 years) are more underweight than older mothers (20–49 years) in Afghanistan, attributing this to increased mother-child nutritional demands. Some previous research showed an association between lower socioeconomic status and underweight [18, 35, 36], but this study did not find this. One possible reason for this nonsignificant finding may be related to the measurement of economic status, which in this study was limited to the number of adult household members.

In bivariate analysis, obesity was higher in women (23.7%) compared to men (12.1%), which is in line with previous studies [12, 35, 36]. Consistent with previous research [14, 35, 36], overweight/obesity increased with age. While some previous studies [12, 35–37] found an association between higher economic status (less household crowding), education, and residing in urban areas and having overweight/obesity, this survey did not show significant associations. Similar results of a non-association between education, income, and job categories with overweight/obesity in adults in Kabul [14]. It is possible that educational level did not impact on body weight status because of the high proportion of the study population (59.0%) not having a formal education. Of concern is as well that 32.3% of young people aged 18–29 years were already overweight or obese, showing that a large proportion of overweight/obesity is already established in early adulthood. Therefore, obesity interventions starting in childhood or adolescents should be prioritized in Afghanistan [38].

In terms of dietary behaviour, previous research found associations between inadequate fruit and vegetable intake [39, 40], eating occasions away from home [41], high salt intake [42], consumption of processed foods and saturated fat [43], and obesity, while this study did not find significant associations between dietary behaviour (inadequate fruit and vegetable intake, having meals outside home, high salt intake, high intake of processed foods, and intake of saturated fats) and underweight as well as overweight or obesity. This study lacked to assess other dietary behaviours, such as frequent snacking, skipping breakfast, and high intake of sugary beverages, which may have been responsible for a higher rate of overweight/obesity [3, 44].

In agreement with previous studies [37–39, 45, 46], this study showed that physical activity was inversely and high sedentary behaviour was in bivariate analysis positively associated with overweight/obesity. Unlike some previous research [12, 38, 45], this study showed no (negative) association between current tobacco use and the prevalence of overweight/obesity. Consistent with previous research in Laos [33], this study found that current tobacco use increased and having hypertension decreased the odds of having underweight. As shown previously for hypertension [10, 12] and diabetes [10, 12, 38], we found an association between NCDs (hypertension and diabetes) and overweight/obesity. This result emphasizes the fact that adults in Afghanistan suffer from several NCD risk factors at the same time [13], calling for multiple risk factor interventions [12, 14].

### Study limitations

Apart from physical and biomedical measures self-reported questionnaire data may have suffered from biased responses. Another limitation was the cross-sectional nature of the survey, which does not allow for causative conclusions. Some variables, such as more details on dietary behaviour, should be included in future studies.

## Conclusion

The study found in the 2018 adult national Afghanistan STEPS survey that almost one in ten adult participants were underweight and more than two in five were overweight/obese. Several factors associated with body weight status, including female sex, current tobacco use and sedentary behaviour for underweight and older age, hypertension, type 2 diabetes, and physical inactivity for overweight/obesity, were identified. Increased public health interventions are needed to address both forms of malnutrition (underweight and overweight/obesity) and associated factors to improve the health of Afghans.

## Data Availability

“The data for the current study are publicly available at the World Health Organization NCD Microdata Repository (URL: https://extranet.who.int/ncdsmicrodata/index.php/catalog).”

## Declarations

All methods were carried out in accordance with relevant guidelines and regulations.
